# Supplementary material for: BNIP3 phosphorylation by JNK1/2 promotes mitophagy via enhancing its stability under hypoxia
Source: Cell Death Dis. 2022 Nov 17;13(11):966. doi: 10.1038/s41419-022-05418-z (PMC9672126; doi:10.1038/s41419-022-05418-z)
Supplement: Supplementary file 1 — SUPPLEMENTAL MATERIAL [file 41419_2022_5418_MOESM1_ESM.pdf]

Supplementary material for

BNIP3 phosphorylation by JNK1/2 promotes mitophagy via enhancing its stability under hypoxia

Yun-Ling He, Jian Li, Sheng-Hui Gong, Xiang Cheng, Ming Zhao, Yan Cao, Tong Zhao, Yong-Qi Zhao, Ming Fan, Hai-Tao Wu, Ling-Ling Zhu, Li-Ying Wu

Corresponding authors:

Ming Fan, Email: fanmingchina@126.com;

Hai-Tao Wu, Email: wuht@bmi.ac.cn;

Ling-Ling Zhu, Email: linglingzhu@hotmail.com;

Li-Ying Wu, Email: liyingwu_china@163.com

This file includes:

Fig. S1. Hypoxia regulates mitophagy and phosphorylation of BNIP3.
Fig. S2. Phosphorylation of BNIP3 at S60/T66 is critical to promote mitophagy
Fig. S3 BNIP3 interacts with ubiquitin.
Fig. S4. JNK1/2 is the kinase responsible for BNIP3 phosphorylation.
Fig. S5. BNIP3 phosphorylation at S60/T66 by JNK enhances mitophagy.
Table S1. The primer information for Site-directed mutagenesis and siRNA-resistant constructs.
Table S2. List of siRNA used in this study.
Table S3. Sequence of real-time PCR primer used in this study.

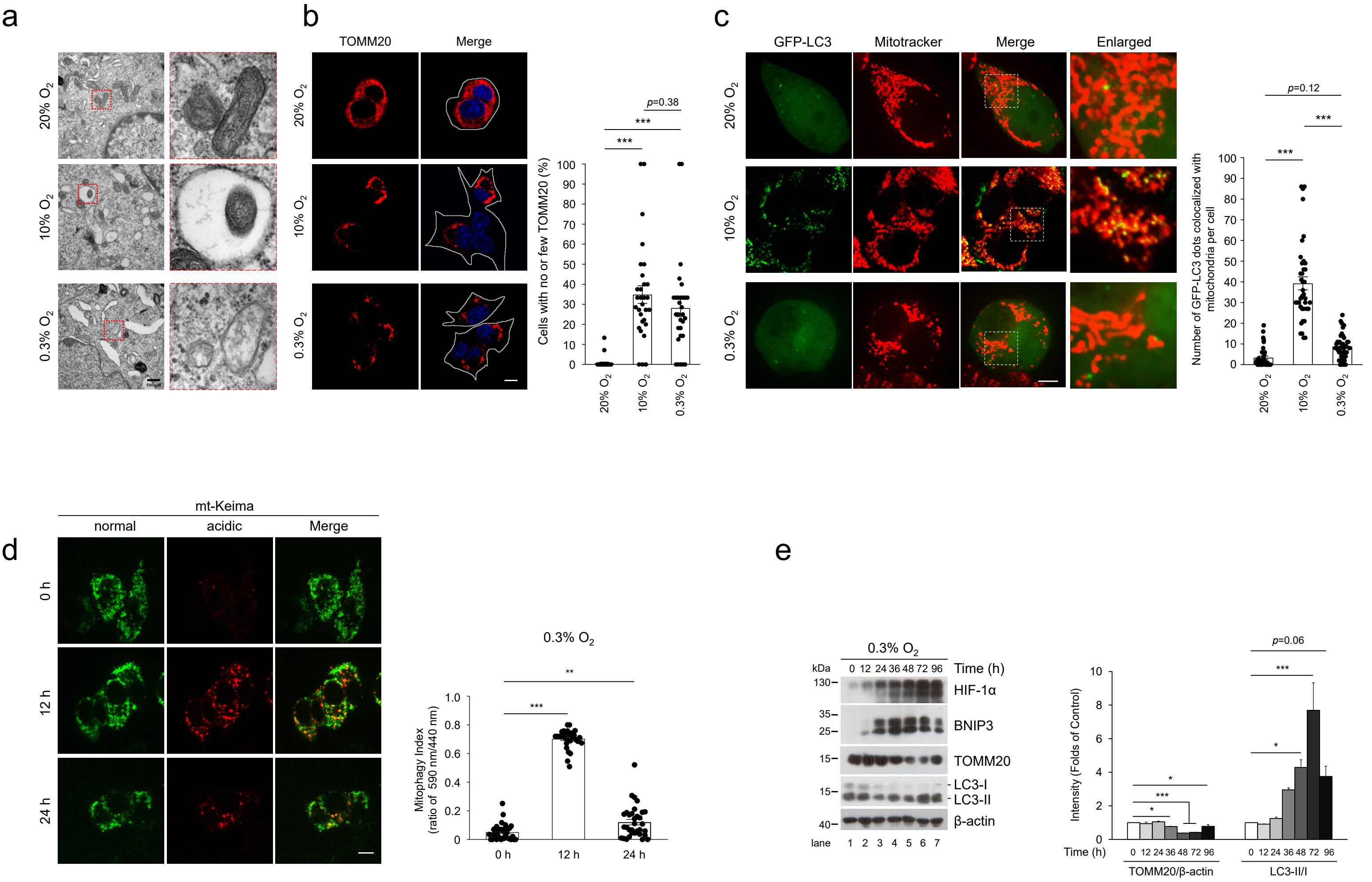

a
20% O2
10% O2
0.3% O2
b
TOMM20
Merge
20% O2
10% O2
0.3% O2
Cells with no or few TOMM20 (%)
p=0.38
***
***
c
GFP-LC3
Mitotracker
Merge
Enlarged
20% O2
10% O2
0.3% O2
Number of GFP-LC3 dots colocalized with mitochondria per cell
p=0.12
***
***
mt-Keima
normal
acidic
Merge
d
0 h
12 h
24 h
0.3% O2
Mitophagy Index (ratio of 590 nm/440 nm)
**
***
e
0.3% O2
kDa
Time (h)
0 12 24 36 48 72 96
130
35
25
15
15
40
HIF-1α
BNIP3
TOMM20
LC3-I
LC3-II
β-actin
lane
1 2 3 4 5 6 7
Intensity (Folds of Control)
p=0.06
***
*
*
***
*
Time (h)
TOMM20/β-actin
LC3-II/I

Fig. S1. Hypoxia regulates mitophagy and phosphorylation of BNIP3.

(a) Mitochondria morphology was analyzed via transmission electron microscopy (TEM) after PC12 cells were exposed to 20% O_2, 10% O_2 or 0.3% O_2 for 24 h. The red boxes indicate representative mitochondria exposed to different oxygen conditions. Scale bar, 500 nm. (b) PC12 cells were exposed to 20% O_2, 10% O_2 or 0.3% O_2 for 24 h. Cells were identified by immunofluorescence staining with antibodies against β-actin (white outline) and TOMM20 (red), and nuclear DNA was marked using DAPI (blue). Scale bars, 10 µm. The percentage of cells with no or few TOMM20 was quantified. $n = 30$. (c) PC12 cells were treated the same as in **a**, and mitophagy was identified and quantified by co-localization of autophagosomes (GFP-LC3, green) and mitochondria (Mitotracker, red). Scale bar, 10 µm. $n = 40$. (d) PC12 cells stably expressing mt-Keima were exposed to 0.3% O_2 for 0, 12, 24 h, and mitophagy was quantified by the ratio of acidic (590 nm, red) to normal mitochondria (440 nm, green). Scale bar, 10 µm. $n \geqq 30$. (e) HeLa cells were exposed to 0.3% O_2 for the indicated time, the levels of BNIP3 and mitophagy related proteins were detected via western blotting. $n=3$. The data are expressed as means ± SEM. $^{*}P < 0.05$, $^{**}P < 0.01$, $^{***}P < 0.001$ versus the indicated group.

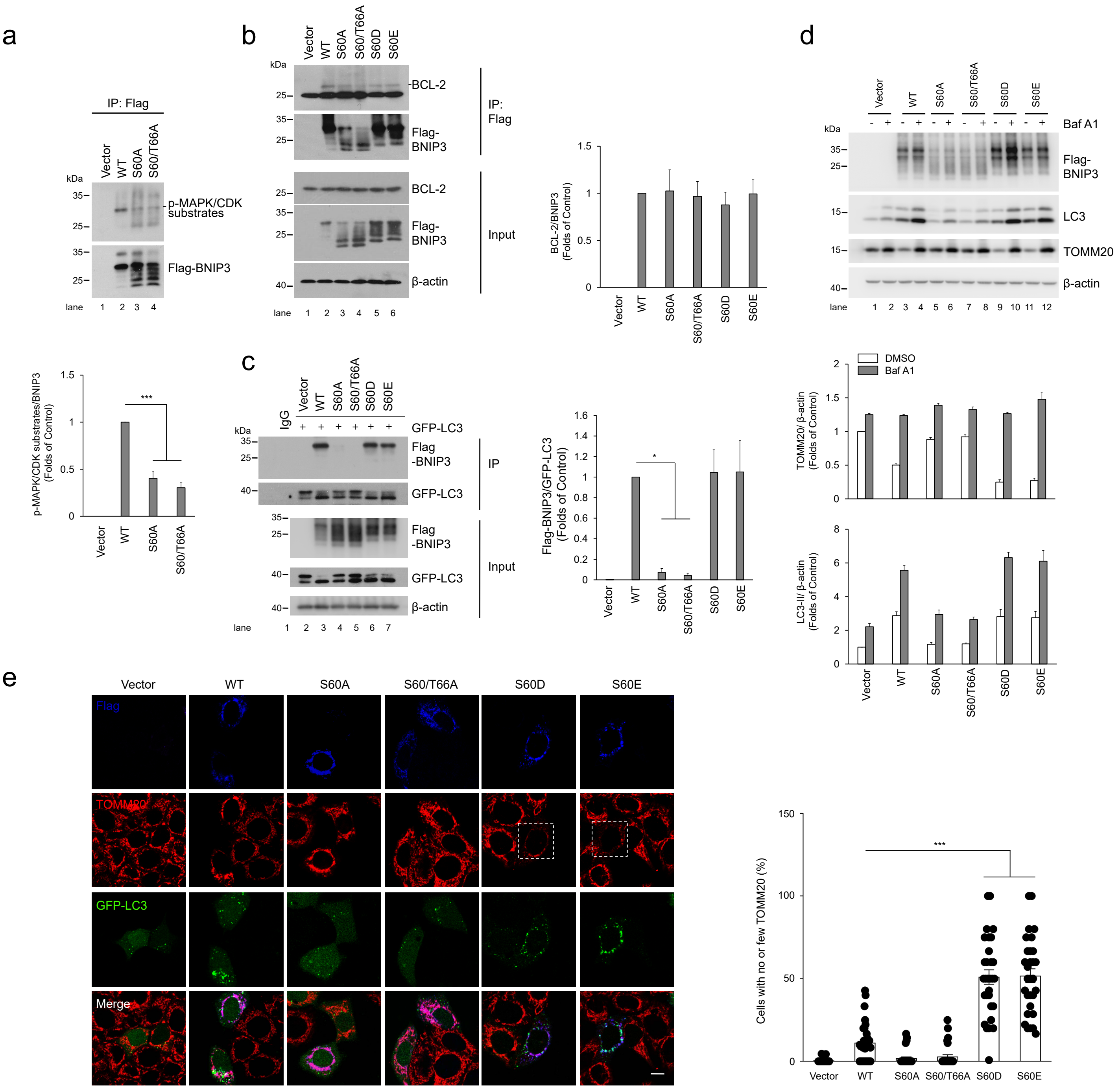
a
IP: Flag
Vector
WT
S60A
S60/T66A
kDa
35
25
p-MAPK/CDK substrates
Flag-BNIP3
lane 1 2 3 4
p-MAPK/CDK substrates/BNIP3 (Folds of Control)
***
b
Vector
WT
S60A
S60/T66A
S60D
S60E
BCL-2
Flag-BNIP3
IP: Flag
Input
β-actin
lane 1 2 3 4 5 6
BCL-2/BNIP3 (Folds of Control)
c
IgG
GFP-LC3
Flag -BNIP3
IP
Input
lane 1 2 3 4 5 6 7
Flag-BNIP3/GFP-LC3 (Folds of Control)
*
d
Baf A1
LC3
TOMM20
lane 1 2 3 4 5 6 7 8 9 10 11 12
DMSO
Baf A1
TOMM20/ β-actin (Folds of Control)
LC3-II/ β-actin (Folds of Control)
e
Flag
TOMM20
GFP-LC3
Merge
Cells with no or few TOMM20 (%)

Fig. S2. Phosphorylation of BNIP3 at S60/T66 is critical to promote mitophagy.

(a) HeLa cells were transfected with empty vector, WT or the indicated mutated Flag-BNIP3, and then, the cell lysates were subjected to immunoprecipitation (IP) with an anti-Flag antibody. Phosphorylation of BNIP3 at S60/T66 was detected in the immune complexes via western blotting using an anti-phospho-MAPK/CDK substrates antibody. $n = 3$. (b) HeLa cells were transfected with empty vector, WT or the indicated Flag-BNIP3 mutants for 48 h. Cell lysates were immunoprecipitated with the anti-Flag antibody and then subjected to western blot analysis with BCL-2 and Flag antibodies. $n = 3$. (c) HeLa cells were co-transfected with GFP-LC3 and empty vector, WT or the indicated Flag-BNIP3 mutants for 48 h, same as Figure 2g, cell lysates were immunoprecipitated with the anti-mouse IgG or the anti-GFP antibody and detected by western blotting. $n = 3$. (d) HeLa cells were transfected with empty vector, WT or the indicated Flag-BNIP3 mutants for 48 h and treated with 20 nM bafilomycin A1 (Baf A1) for additional 12 h. Cell lysates were analyzed by western blotting. $n = 3$. (e) HeLa cells were transfected with GFP-LC3 and empty vector, WT or the indicated Flag-BNIP3 mutants for 48 h. The cells were then fixed and immunostained with Flag (blue) and TOMM20 (Red). The white boxes indicate cells with few mitochondria. Scale bars, 10 μm. $n = 30$. The data are expressed as means ± SEM. $^{*}P < 0.05$, $^{**}P < 0.01$, $^{***}P < 0.001$ versus the indicated group.

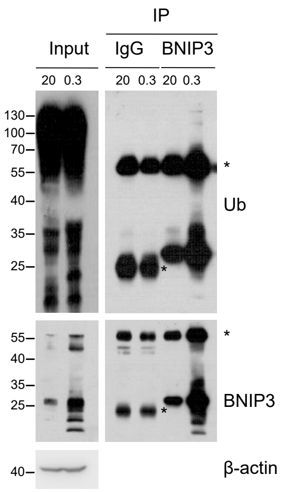

* IgG heavy and light chains

Fig. S3 BNIP3 interacts with ubiquitin.

PC12 cells were exposed to 20% O_2 or 0.3% O_2 supplemented with 10 μM MG132 for 6 h. Cell lysates were boiled and immunoprecipitated with mouse IgG or anti-BNIP3 antibody. Then, the immune complexes were analyzed via western blotting. Asterisks indicate the heavy and light chains of IgG.

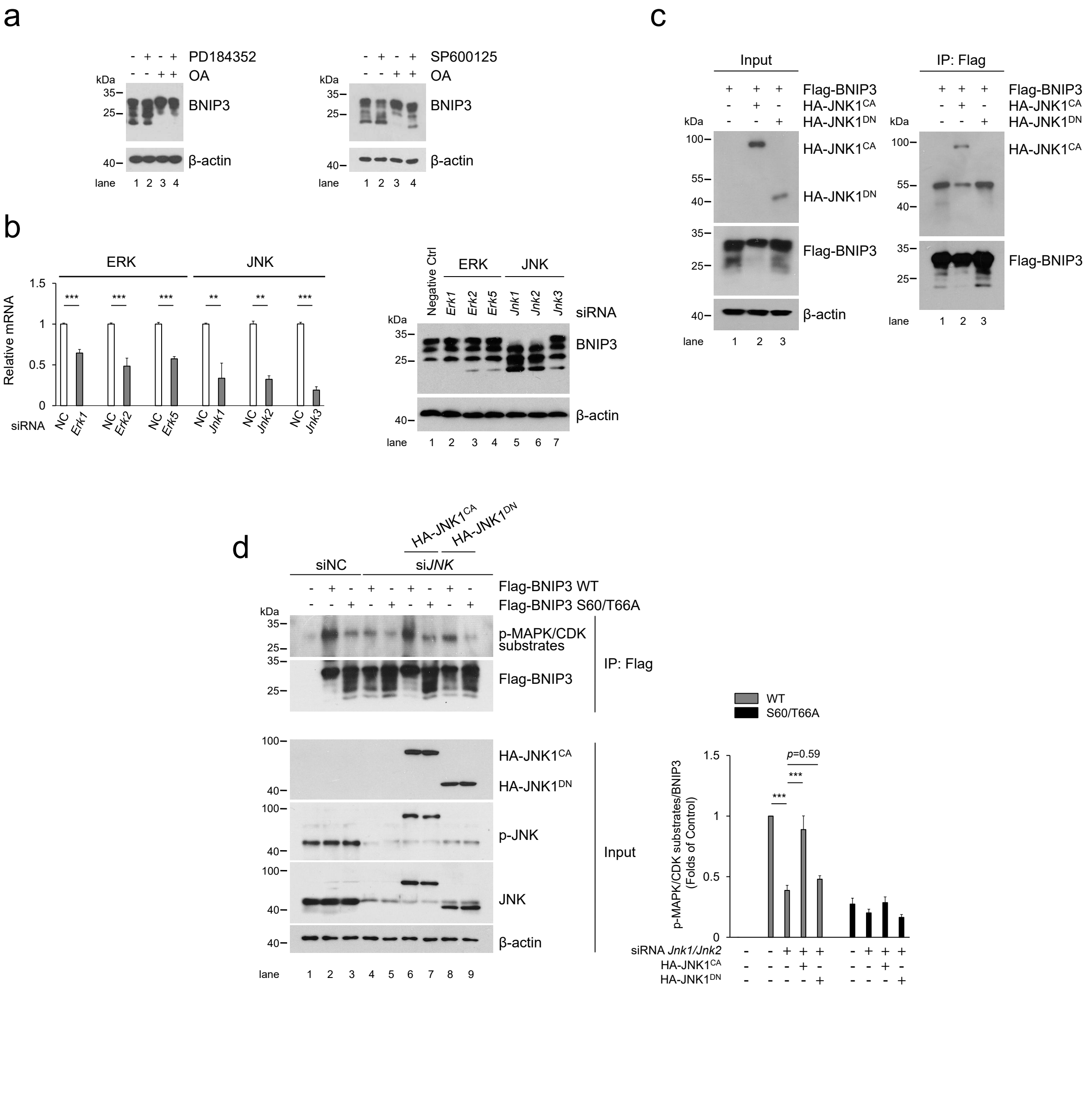
a
PD184352
OA
kDa
35
25
40
BNIP3
β-actin
lane 1 2 3 4
SP600125
OA
BNIP3
β-actin
lane 1 2 3 4
b
ERK
JNK
1.5
1
0.5
0
Relative mRNA
*** *** *** ** ** ***
siRNA NC Erk1 NC Erk2 NC Erk5 NC Jnk1 NC Jnk2 NC Jnk3
Negative Ctrl
ERK
JNK
Erk1 Erk2 Erk5 Jnk1 Jnk2 Jnk3
siRNA
BNIP3
β-actin
lane 1 2 3 4 5 6 7
c
Input
IP: Flag
Flag-BNIP3
HA-JNK1^CA
HA-JNK1^DN
kDa
100
55
40
35
25
HA-JNK1^CA
HA-JNK1^DN
Flag-BNIP3
β-actin
lane 1 2 3
d
HA-JNK1^CA
HA-JNK1^DN
siNC
siJNK
Flag-BNIP3 WT
Flag-BNIP3 S60/T66A
p-MAPK/CDK substrates
Flag-BNIP3
IP: Flag
HA-JNK1^CA
HA-JNK1^DN
p-JNK
JNK
β-actin
Input
lane 1 2 3 4 5 6 7 8 9
WT
S60/T66A
p-MAPK/CDK substrates/BNIP3 (Folds of Control)
1.5
1
0.5
0
p=0.59
***
***
siRNA Jnk1/Jnk2
HA-JNK1^CA
HA-JNK1^DN

Fig. S4. JNK1/2 is the kinase responsible for BNIP3 phosphorylation.

(a) PC12 cells were treated with okadaic acid (OA) and PD184352 (left) or SP600125 (right) for 12 h and analyzed via western blotting with an anti-BNIP3 or anti-β-actin antibody. (b) PC12 cells were transfected with negative control (NC) or the indicated siRNA for 48 h, and the mRNA levels of related genes (left) and BNIP3 expression (right) were detected by real-time PCR and western blotting, respectively. $n \geqq 3$. (c) HeLa cells were transfected with Flag-BNIP3 and constitutively active JNK1 (HA-JNK1CA) (HA-MKK7-JNK1) or dominant negative JNK1 (HA-JNK1DN) (HA-JNK1-APF) for 48 h. Cell lysates were then immunoprecipitated with an anti-Flag antibody and detected by western blotting with an anti-HA or anti-Flag antibody. (d) *JNK1* and *JNK2* knockdown HeLa cells were transfected with WT or S60/T66A and HA-JNK1^{CA} or HA-JNK1^{DN} mutants, and 48 h post-transfection, cell lysates were immunoprecipitated with an anti-Flag antibody. The immune complexes were then analyzed via western blotting with the indicated antibodies. $n = 3$. The data are expressed as means ± SEM. *$P < 0.05$, **$P < 0.01$, ***$P < 0.001$ versus the indicated group.

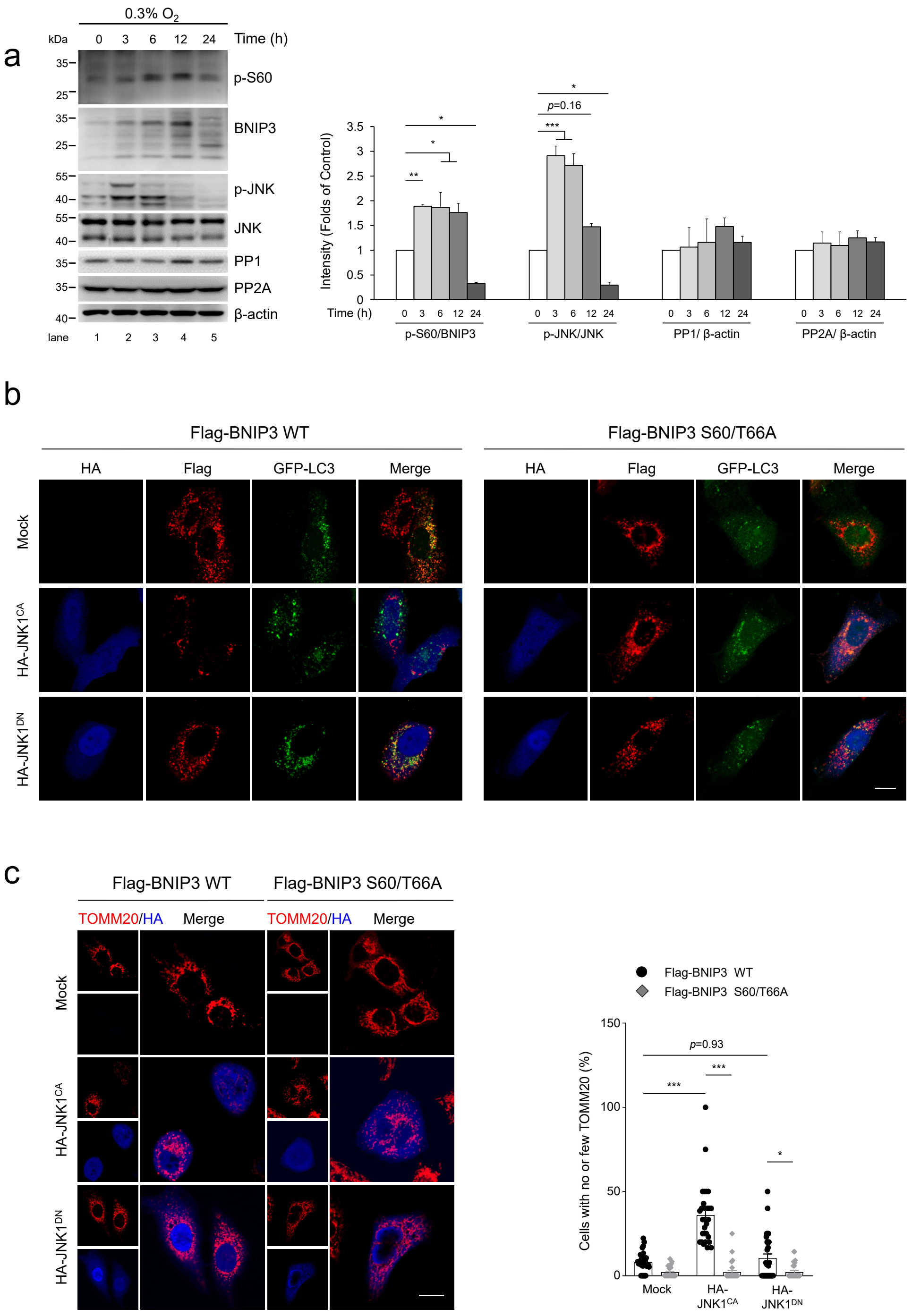

a
0.3% O2
kDa
0 3 6 12 24
Time (h)
p-S60
BNIP3
p-JNK
JNK
PP1
PP2A
β-actin
lane
1 2 3 4 5
Intensity (Folds of Control)
Time (h)
p-S60/BNIP3
p-JNK/JNK
PP1/ β-actin
PP2A/ β-actin
p=0.16
b
Flag-BNIP3 WT
Flag-BNIP3 S60/T66A
HA
Flag
GFP-LC3
Merge
Mock
HA-JNK1CA
HA-JNK1DN
c
Flag-BNIP3 WT
Flag-BNIP3 S60/T66A
TOMM20/HA
Merge
Flag-BNIP3 WT
Flag-BNIP3 S60/T66A
Cells with no or few TOMM20 (%)
p=0.93
Mock
HA-JNK1CA
HA-JNK1DN

Fig. S5. BNIP3 phosphorylation at S60/T66 by JNK enhances mitophagy.

(a) PC12 cells were exposed to 0.3% O_2 for the indicated time, phosphorylation of BNIP3 and expression of PP1 and PP2A and JNK activity were detected via western blotting. *n* = 3. (b) Representative images of GFP-LC3 puncta in Flag-BNIP3 stably expressed HeLa cells, co-transfected with plasmids encoding HA-JNK1. Cells were identified by immunofluorescence staining with antibodies against HA (blue) and Flag (red). Scale bars, 10 μm. (c) HeLa cells stably expressing Flag-BNIP3 were transfected with CA or DN HA-JNK1 and immunostained with HA (blue) and TOMM20 (Red). The percentage of cells with no or few TOMM20 was quantified. Scale bars, 10 μm. *n* = 30. The data are expressed as means ± SEM. **P* < 0.05, ***P* < 0.01, ****P* < 0.001 versus the indicated group.

Table S1. The primer information for the site-directed mutagenesis and the siRNA-resistant constructs.

**Plasmids name**	**Forward primer**	**Reverse primer**
S12A	5’-AGAACCTGCAGGGCGCCTGGGTAGAACTGC-3’	5’-GCAGTTCTACCCAGGCGCCCTGCAGGTTCT-3’
S19A	5’-TAGAACTGCACTTCGCCAATGGGAATGGGA-3’	5’-TCCCATTCCCATTGGCGAAGTGCAGTTCTA-3’
S48A	5’-ATGCGCAGCATGAAGCTGGACGAAGCAGCT-3’	5’-AGCTGCTTCGTCCAGCTTCATGCTGCGCAT-3’
S56A	5’-GCAGCTCCAAGAGCGCTCACTGTGACAGCC-3’	5’-GGCTGTCACAGTGAGCGCTCTTGGAGCTGC-3’
S60A	5’-GCTCTCACTGTGACGCCCCACCTCGCTCCC-3’	5’-GGGAGCGAGGTGGGGCGTCACAGTGAGAGC-3’
S60D	5’-GCTCTCACTGTGACGACCCACCTCGCTCCC-3’	5’-GGGAGCGAGGTGGGTCGTCACAGTGAGAGC-3’
S60E	5’-GCTCTCACTGTGACGAACCACCTCGCTCCC-3’	5’-GGGAGCGAGGTGGTTCGTCACAGTGAGAGC-3’
T66A	5’-CACCTCGCTCCCAGGCACCACAAGATACCA-3’	5’-TGGTATCTTGTGGTGCCTGGGAGCGAGGTG-3’
S79A	5’-GAAATAGACACCCACGCCTTTGGTGAGAAAAA-3’	5’-TTTTTCTCACCAAAGGCGTGGGTGTCTATTTC-3’
S85/T86A	5’-GTGAGAAAAACGCCGCTCTGTCTGAGGAAG-3’	5’-CTTCCTCAGACAGAGCGGCGTTTTTCTCAC-3’
S88A	5’-GAAAAACAGCACTCTGGCTGAGGAAGATTATA-3’	5’-TATAATCTTCCTCAGCCAGAGTGCTGTTTTTC-3’
T141/S142A	5’-GCATGAGAAACGCAGCCGTGATGAAGAAAG-3’	5’-CTTTCTTCATCACGGCTGCGTTTCTCATGC-3’
Bnip3 siRNA-resistant	5’-TTGGAAGGCGTTTAACGACCAGTACGTCCACCTTTTGAGGATCC-3’	5’-GGATCCTCAAAAGGTGGACGTACTGGTCGTTAAACGCCTTCCAA-3’
Jnk1 siRNA-resistant	5’-TCCTTGGCGAGATGGAGTATAAAGAAAACGTGGA-3’	5’-TCCACGTTTTCTTTATACTCCATCTCGCCAAGGA-3’

Table S2. List of siRNA used in this study.

**Gene name**	**Species**	**Target Sequences**
Bnip3	Rat	5'-CTGACAACTTCCACTAGTA-3'
Erk1 (Mapk3)	Rat	5'-CGGCTGAAGGAGCTGATCT-3'
Erk2 (Mapk1)	Rat	5'-GTGCTGTGTCTTCAAGAGC-3'
Erk5 (Mapk7)	Rat	5'-CCCTCAGGGAACTGAAGAT-3'
Jnk1 (Mapk8)	Rat	5-GCATGGGCTACAAGGAGAA-3'
JNK1 (MAPK8)	Human	5'-AGCTCCACCACCAAAGATC-3'
Jnk2 (Mapk9)	Rat	5'-GGAATTGTTTGTGCTGCTT-3'
JNK2 (MAPK9)	Human	5'-TTCCAAGGCACTGACCATA-3'
Jnk3 (Mapk10)	Rat	5'-GCCTCCGCCTCAGATATAT-3'
Ppp1ca #1	Rat	5'-GCTTGTTGCTGGCCTATAA-3'
Ppp1ca #2	Rat	5'-GAAATAGCCTCCATGTGCT-3'
Ppp1cb #1	Rat	5'-CAAGTCTCGTGAAATCTTT-3'
Ppp1cb #2	Rat	5'-CTTTATGATGTCACACCTT-3'
Ppp1cc #1	Rat	5'-GTGACATCCACGGGCAGTA-3'
Ppp1cc #2	Rat	5'-GTTGAAGATGGATATGAGT-3'
Ppp2ca #1	Rat	5'-GAAAGTTTAACCTTGTACA-3'
Ppp2ca #2	Rat	5'-GATACAAATTACTTGTTTA-3'
Ppp2cb #1	Rat	5'-CTTTGATTATCTTCCACTT-3'
Ppp2cb #2	Rat	5'-GCTTGTAATGGAAGGATAT-3'

Table S3. Sequence of real-time PCR primer used in this study.

Gene name	Species	Forward primer	Reverse primer
Actb	Rat	5’-GCAGGAGTACGATGAGTCCG-3’	5’-ACGCAGCTCAGTAACAGTCC-3’
Bnip3	Rat	5’-GTCGCAGAGCGGGGAGGAGA-3’	5’-TCTGGGAGCGAGGTGGGCTG-3’
Erk1 (Mapk3)	Rat	5’-TACCGAGCCCCAGAGATCAT-3’	5’-TGGGATGGGGAACCCAGTAT-3’
Erk2 (Mapk1)	Rat	5’-AATGTTCTGCACCGTGACCT-3’	5’-TGGTCTGGATCTGCAACACG-3’
Erk5 (Mapk7)	Rat	5’-CGCCCCACCTTTTGACTTTG-3’	5’-CCATGGCACAGTCTCCACTT-3’
Jnk1 (Mapk8)	Rat	5’-ACTTAAAGCCAGTCAGGCGA-3’	5’-TTGATGTACGGGTGCTGGAG-3’
Jnk2 (Mapk9)	Rat	5’-TCCAGAAGTCATCCTGGGCA-3’	5’-CTCTTCCTGGGAACAGGACTT-3’
Jnk3 (Mapk10)	Rat	5’-GTTTGGTACGACCCTGCTGA-3’	5’-GAGGGCTGGCCTTTGACTAC-3’
