# Supplementary figures and images for: BNIP3 phosphorylation by JNK1/2 promotes mitophagy via enhancing its stability under hypoxia
Source: Cell Death Dis. 2022 Nov 17;13(11):966. doi: 10.1038/s41419-022-05418-z (PMC9672126; doi:10.1038/s41419-022-05418-z)

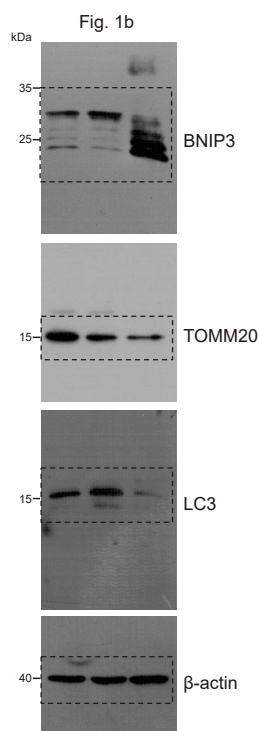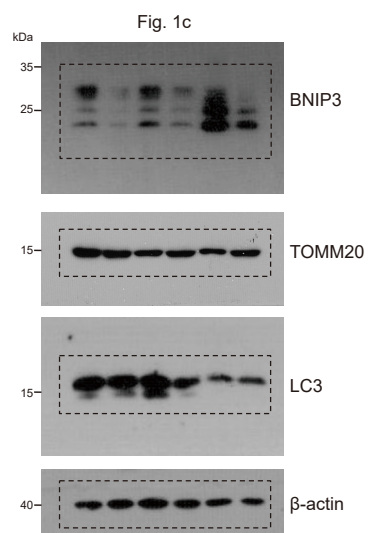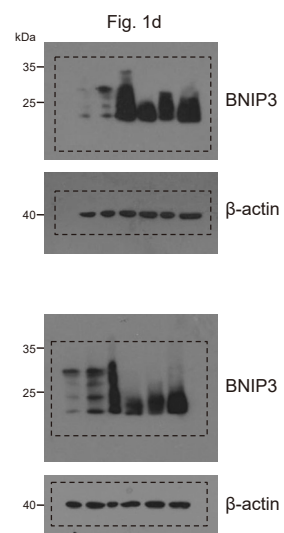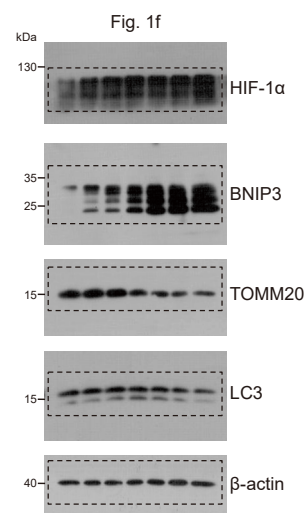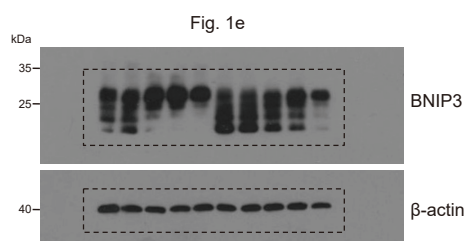

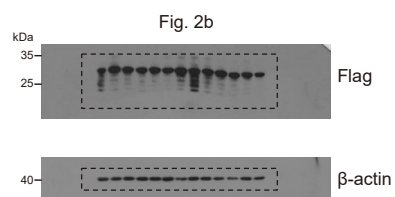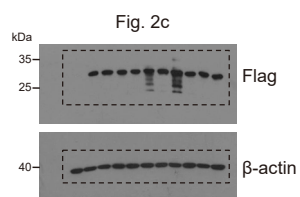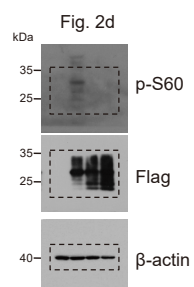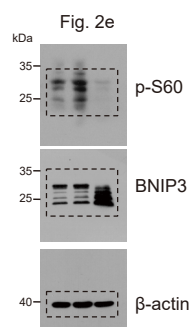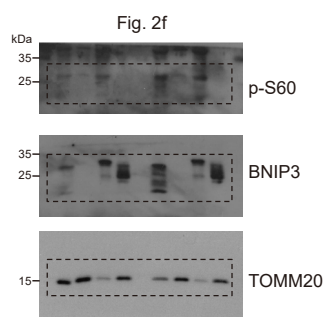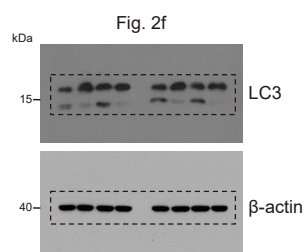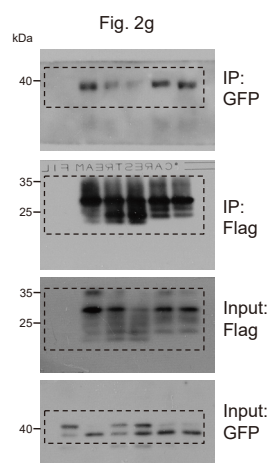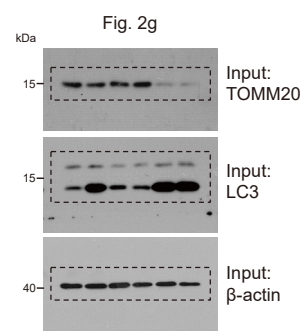

Fig. 3a (left)

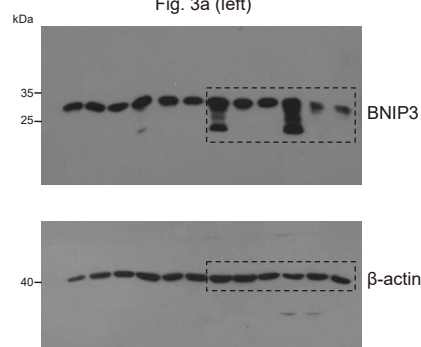

Fig. 3a (right)

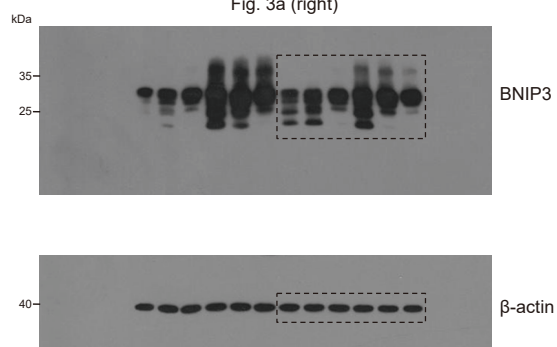

Fig. 3g

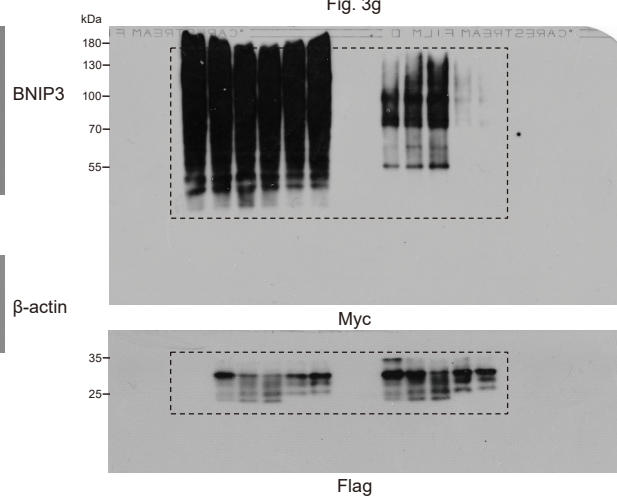

Fig. 3c

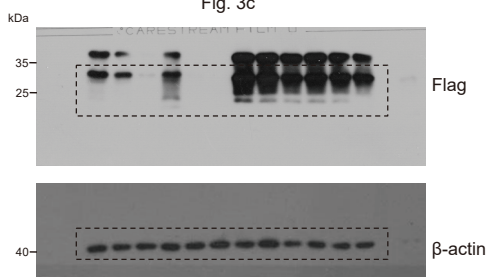

Fig. 3e

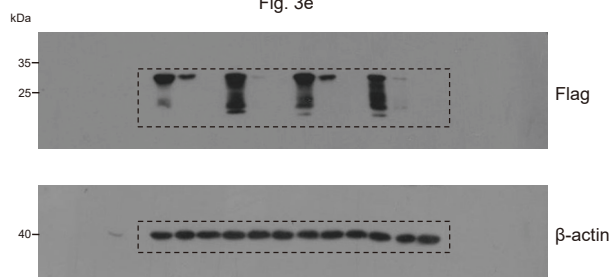

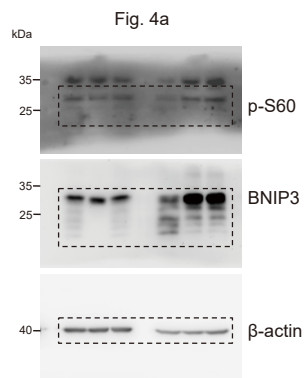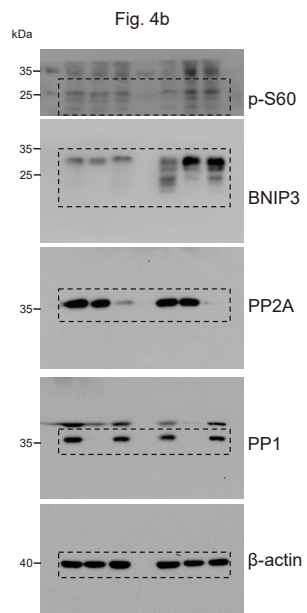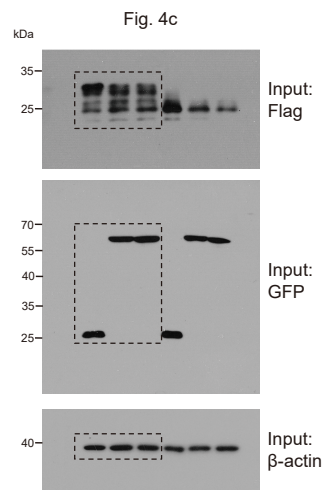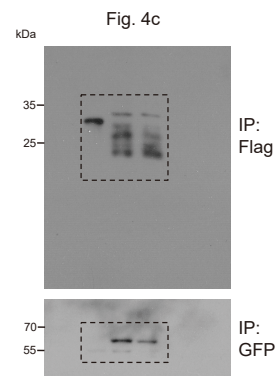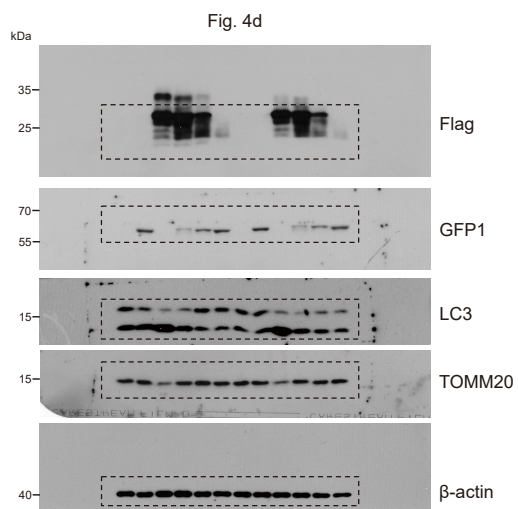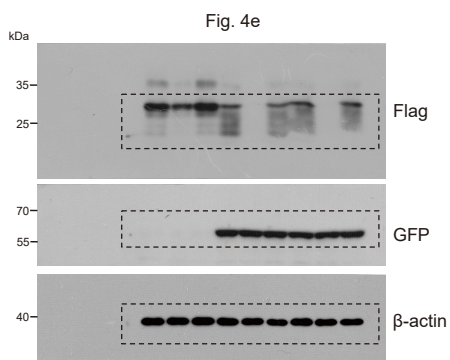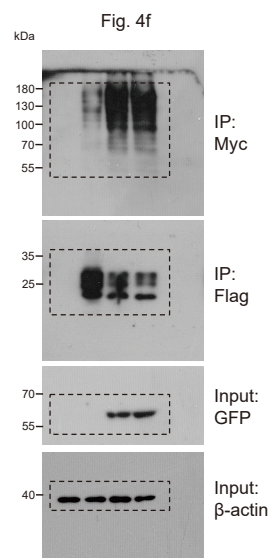

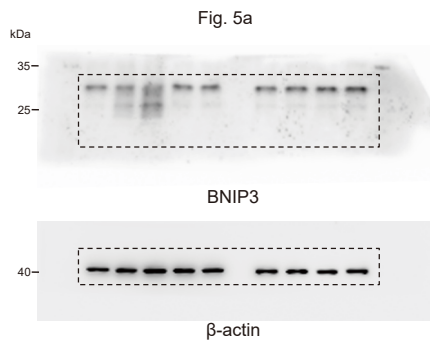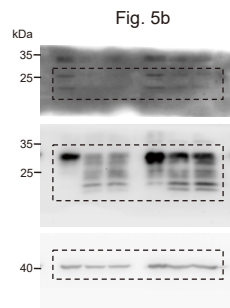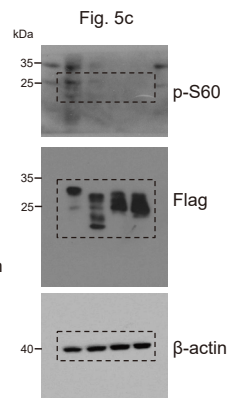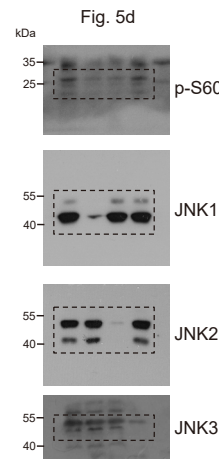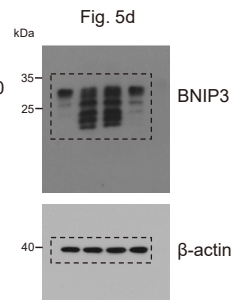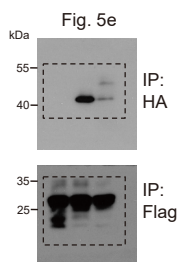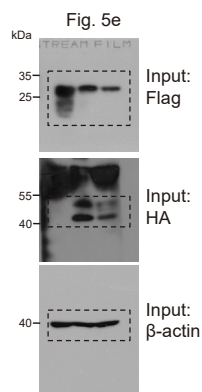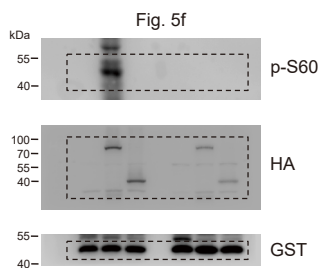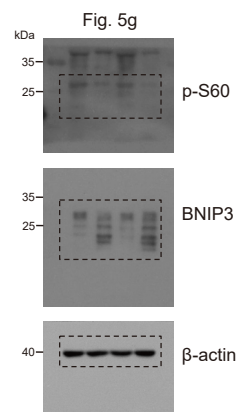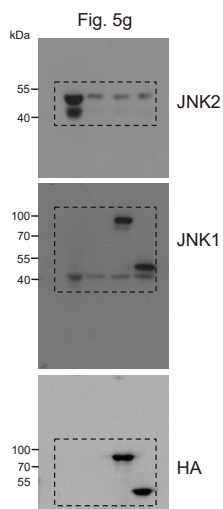

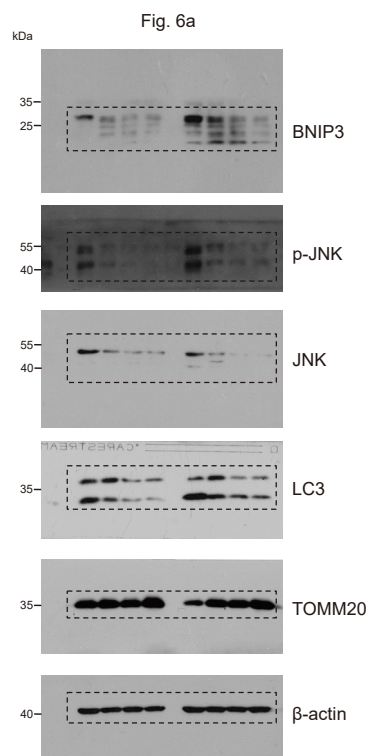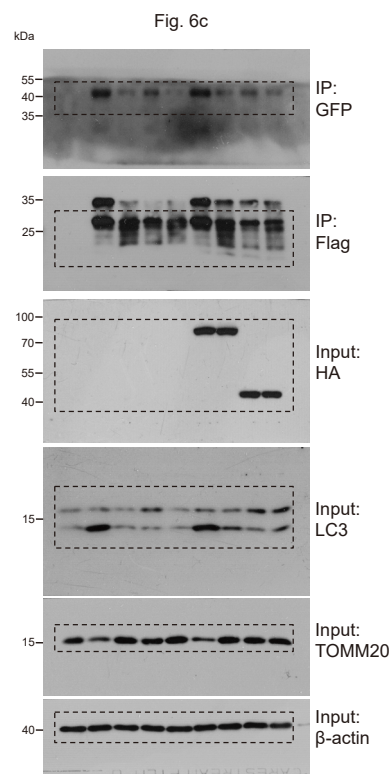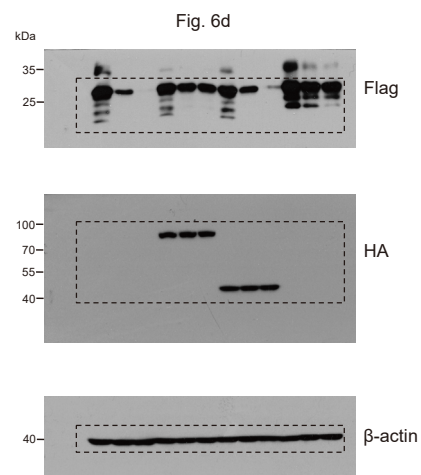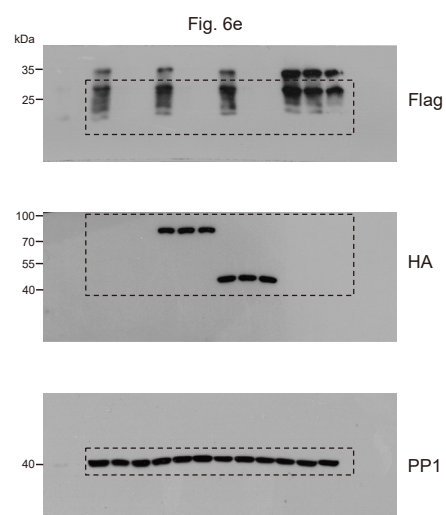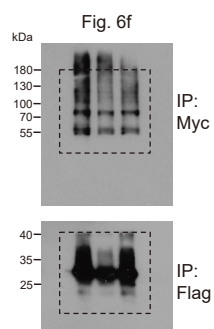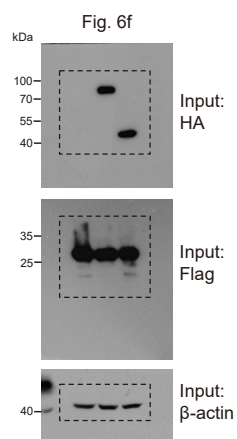

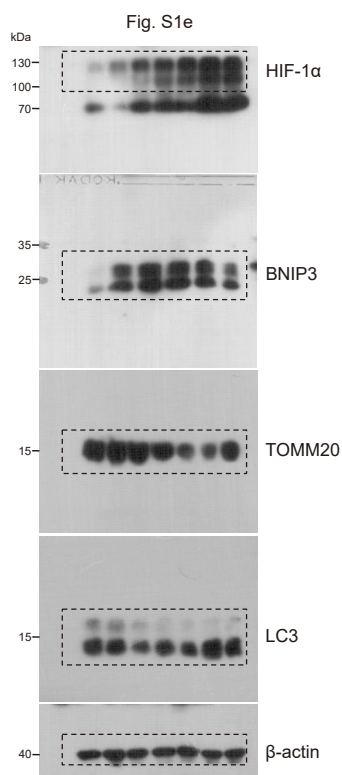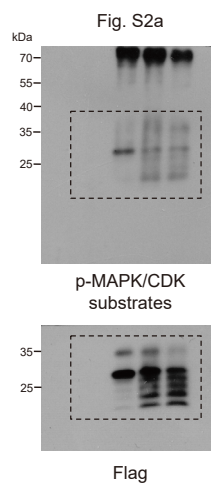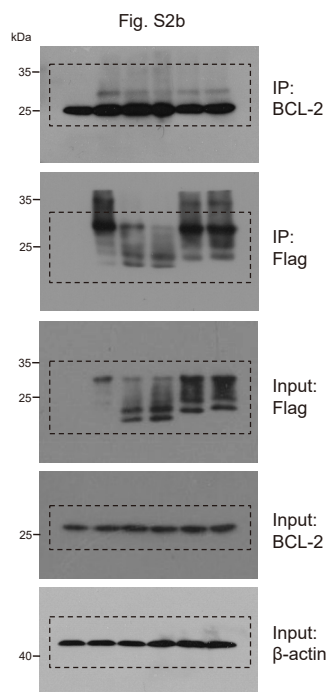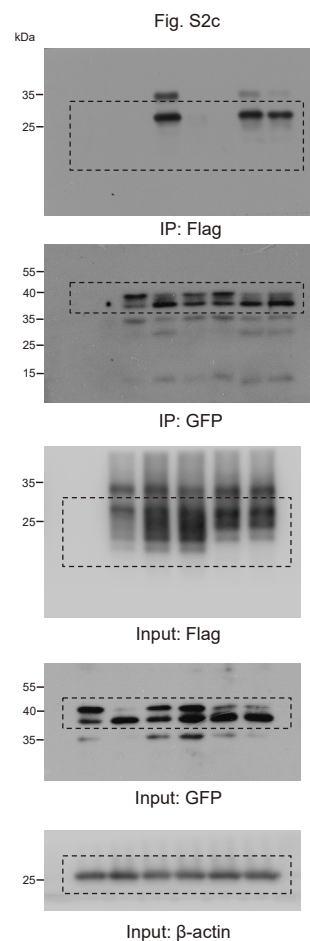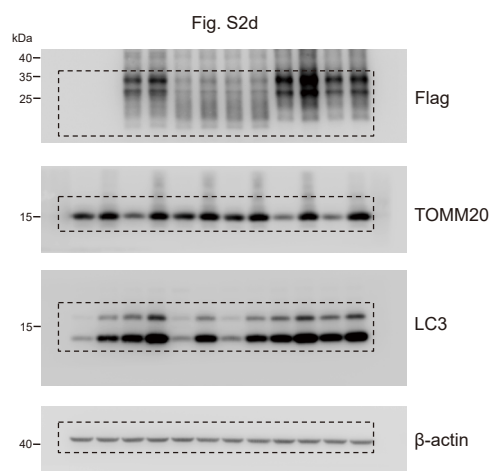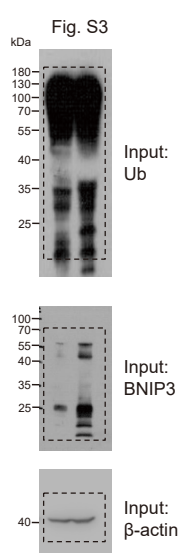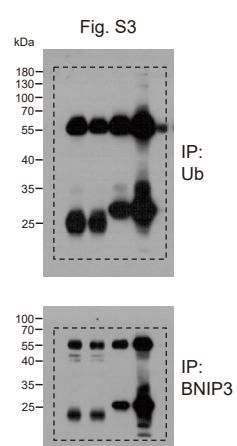

Fig. S4a (left)

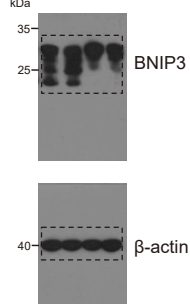

Fig. S4a (right)

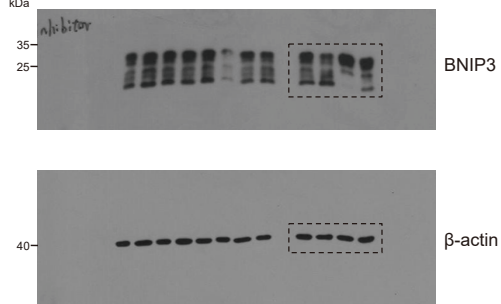

Fig. 4c

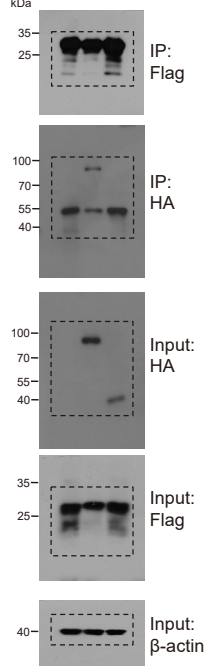

Fig. S4d

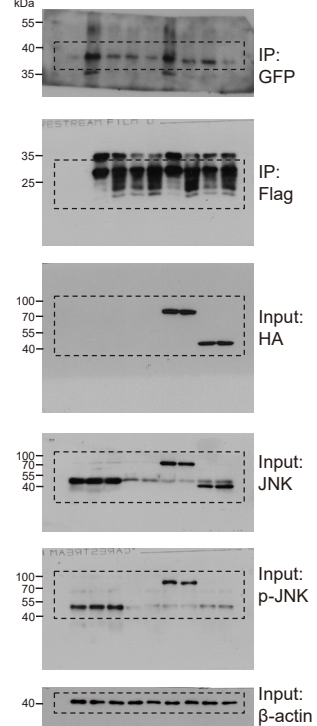

Fig. S4b

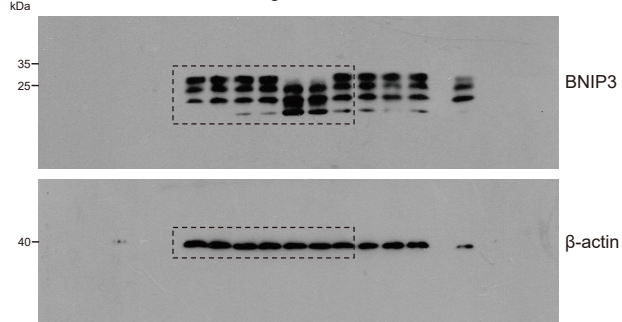

Fig. S5a

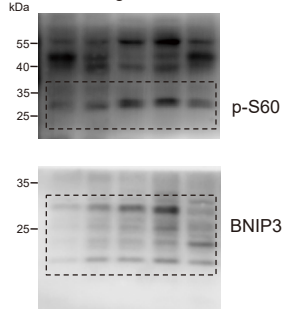

Fig. S5a

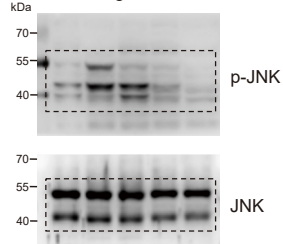

Fig. S5a

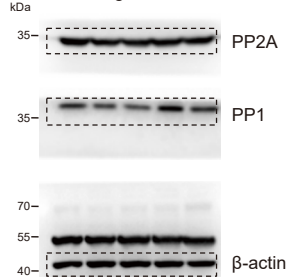

Supplement: Supplementary file 2 — Original Data File [file 41419_2022_5418_MOESM2_ESM.pdf]
